# Comparing the effects of two inhaled glucocorticoids on allergen-induced bronchoconstriction and markers of systemic effects, a randomised cross-over double-blind study

**DOI:** 10.1186/2045-7022-1-12

**Published:** 2011-10-31

**Authors:** Jan Lötvall, Mona Palmqvist, Peter Arvidsson

**Affiliations:** 1 Krefting Research Centre, University of Gothenburg, BOX 424, SE 40530 Göteborg, Sweden

**Keywords:** allergen, asthma, budesonide, mometasone, inflammation

## Abstract

**Background:**

Inhaled glucocorticoids are efficient in protecting against asthma exacerbations, but methods to compare their efficacy vs systemic effects have only been attempted in larger multi-centre studies. The aim of the current study was therefore to directly compare the effects of two separate inhaled glucocorticoids, mometasone and budesonide, to compare the effects on the early and late asthmatic responses to inhaled allergen in patients with mild allergic asthma, and sputum eosinophils, and to relate the clinical positive effects to any systemic effects observed.

**Methods:**

Twelve patients with documented early and late asthmatic responses (EAR and LAR) to inhaled allergen at a screening visit were randomized in a double-blind fashion to treatment with mometasone (200 μg × 2 or 400 μg × 2), budesonide (400 μg × 2) or placebo in a double-blind crossover fashion for a period of seven days. Challenge with the total allergen dose causing both an EAR and LAR was given on the last day of treatment taken in the morning. Lung function was assessed using FEV1, and systemic glucocorticoid activity was quantified using 24 h urinary cortisol.

**Results:**

Mometasone and budesonide attenuate both EAR and LAR to allergen to a similar degree. No significant dose-related effects on the lung function parameters were observed. Both treatments reduced the relative amount of sputum eosinophils (%) after allergen. At the dose of 800 μg daily, mometasone reduced 24 h urinary cortisol by approximately 35%. Both drugs were well tolerated.

**Conclusions:**

Mometasone and budesonide are equieffective in reducing early and late asthmatic responses induced by inhaled allergen challenge. Mometasone 800 μg given for seven days partially affects the HPA axis.

## Background

Asthmatic patients with allergies often develop early (EAR) and in some patients also a late asthmatic response (LAR) when a relevant allergen is inhaled [1-3]. Allergen exposure can also increase non-specific bronchial hyperresponsiveness to stimuli such as methacholine or histamine [4]. The LAR and the bronchial hyperresponsiveness are often associated with increase of eosinophils in the blood, and influx of eosinophils into the airways [3,5].

The anti-inflammatory effects of inhaled glucocorticoids in asthma are well documented [1,6,7], shown by attenuation of the EAR, LAR and allergen-induced sputum eosinophils. Glucocorticoids are also more effective than anti-leukotrienes in attenuating the LARs and improve the bronchial hyperresponsiveness in mild asthmatic patients [8]. Mometasone and budesonide are corticosteroids that have been shown having a significant effect on late events induced by allergen, including attenuation of the LAR and reductions in eosinophil and neutrophil recruitment and activation [5,9,10].

The aim of the present study was to directly compare the effects of inhaled mometasone with inhaled budesonide and placebo on allergen-induced airway responses in mild asthmatic patients. Treatments were given for seven days and the allergen challenge was performed on Day 7 of treatment. Lung function was measured to assess the development of EAR and LAR. Sputum and blood were analysed in order to assess inflammatory processes. To determine whether any differences in systemic effects of the drugs could be detected, urinary cortisol was measured.

## Materials and methods

### Patients

Twelve patients (Table 1) with mild allergic asthma, only occasionally using inhaled beta-2-agonists when need arises, were included in the study. The presence of both an early and a late asthmatic reaction after allergen challenge had to be evident in each patient. That is to say the patients had to develop an early asthmatic reaction, EAR, (a fall in FEV_1 _of at least 20% from baseline within an hour) and a late asthmatic reaction, LAR, (a fall in FEV_1 _of at least 15% from baseline on at least one time point during 4-7 h) after bronchial allergen challenge.

**Table 1:** Characteristics of the patients

**Patient no**.	Sex	Age (years)	**FEV**_**1**_ (L) Baseline	Allergen
1	F	44	2.71	Cat

2	M	33	3.61	Cat

3	M	28	3.56	Cat

4	F	31	2.70	Cat

5	F	43	2.81	Cat

6	F	20	3.46	Cat

7	M	30	3.60	Cat

8	F	26	3.59	Cat

9	M	36	3.70	Cat

10	F	34	2.28	Equine

11	M	27	3.81	Canine

12	M	40	3.96	Cat

Exclusion criteria were inability to produce sputum after inhalation of hypertonic saline, ongoing treatment with inhaled or oral glucocorticoid or long-acting inhaled β-2-agonist, ongoing smoker and pregnancy. Patients with other significant diseases were also excluded.

### Study design

This was a randomized, double-blind, placebo-controlled, cross-over study at one single centre. Approval was obtained by the Swedish Medical Product Agency and the Local Ethics Committee. Written informed consent was received form all patients before enrolment. All patients received four different interventions including mometasone 2 × 200 μg, mometasone 2 × 400 μg, budesonide 2 × 400 μg and placebo with one week wash-out between treatments. The study was performed during 2001-2003, before the requirement to register clinical trials in clinicaltrials.gov.

**Figure 1:**
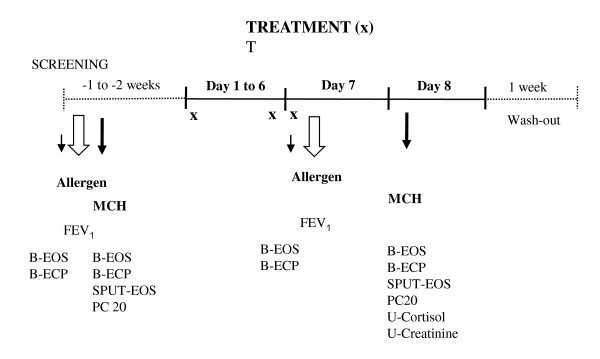
**Schedule for each treatment period**. Treatments were taken each morning and evening, and in the case of mometasone 400 μg, only once daily.

The treatment and challenge protocol is described in Figure 1. All patients were screened during two days. At the screening visit a bronchial allergen challenge was performed. Twenty-four hours later the methacholine responsiveness was tested just prior to the sputum induction. If all inclusion/exclusion criteria were met the patient was randomised on the second day.

The study medication for one treatment period was brought home by the patient and the study treatment was initiated after a telephone contact with the patient one to two weeks after randomisation. Study medications were given as one dose in the morning and evening for 7 days (only in the morning on Day 7).

The patient returned to the clinic on day 7. The cumulative allergen dose titrated on the screening day was given as a single dose allergen challenge (after the morning dose of inhaled treatment) in each treatment period. Induction of sputum was performed 24 h after allergen challenge.

### Allergen challenge

Allergen extracts (cat, dog, or horse) from Copenhagen Allergology Laboratory (ALK), Copenhagen, Denmark, were used. The screening allergen provocation protocol started with the diluents, and the pre titrated allergen dose given was 32 standard quality units (SQU/ml). There was a twofold increase between every dose step. The highest allergen dose was 64 000 SQ-U/ml. FEV_1 _was measured 5 and 10 min after each dose (the best of two manoeuvres with 1 min between). When FEV_1 _had fallen at least 20% from the baseline value, the allergen provocation was stopped (EAR). After the last allergen dose FEV_1 _was measured at 15, 20, 30, 45 and 60 min during the first hour, and then every hour up to 10 h. The cumulative allergen dose titrated on the screening day was given as a single dose on day 7 in all four treatment periods. The FEV_1 _was measured at the same time-points as above.

The LAR was defined as a fall in FEV_1 _of 15% from baseline value on at least one time point between 3 and 7 h after the allergen dose that caused the EAR.

### Methacholine responsiveness

Methacholine challenge was performed 24 h after the allergen challenge at the screening and on Day 8. At a starting concentration of 0.03 mg/ml, twofold increasing concentrations were administered every fifth minute. FEV_1 _was measured at 90 and 180 s after the inhalations. When FEV_1 _had fallen with ≥ 20%, the provocation was stopped (Provocative Concentration changing FEV_1 _by 20% = PC20).

### Bronchial challenge

The dosimeter ME.FAR MB3 (Mefar, Brescia, Italy) was used for all bronchial challenges (allergen and methacholine). The patient inhaled slowly the dose of aerosol by means of an inspiratory capacity breath, followed by 5 s of breath holding. For each dose of allergen or methacholine, five inhalations were given. The nebulization time was set to 1 s. The patients were instructed to use a home spirometer (One Flow soft 1.0 from STI) for the FEV_1 _measurements 1 h to 10 h after allergen exposure.

### Sputum induction

Sputum induction was performed using a modification of the method of Pin et al. [11] with an aerosol of hypertonic saline. Inhalations started with 3% concentrations of saline for 7 min. If FEV_1 _had dropped less than 10% from baseline, the next saline concentration (4 and 5%, respectively) was administered. If the fall in FEV_1 _was between 10 and 20% from baseline, the same saline concentration was given for the next 7 min. The nebulization was stopped if the fall in FEV_1 _was more than 20% from baseline. The sputum was coughed up into a container and further analysed. After the sputum induction at the screening and on day eight, a single oral dose of 30 mg of prednisolone was given to reverse any remaining effects on LAR, as used previously [8].

### Sputum cell analysis

Sputum samples were examined within 2 h (kept cold) using a modified method described by Pizzichini et al.1996 [12-14]. Sputum cells were prepared as described earlier and the total number of cells and cell viability was assessed [8]. Percentage of eosinophils (%) were identified by differential counts using a combination of luxol fast blue and chloroacetate esterase staining.

### Other analyses

Samples for blood eosinophils and ECP were collected at the screening and on day 7 both before and 24 h after allergen challenge. Urinary samples for analysing cortisol and creatinine analyses were collected 24 h post allergen (Day 8).

### Statistics

Power calculations showed that less than five patients are required to document a 50% reduction of LAR with 90% power [15,16]. To document a similar reduction of sputum eosinophils (%), a similar number of patients are required [17]. Therefore, a study including twelve patients further increases the statistical power. Comparisons were made using student's paired t-test, and a p-value < 0.05 was considered statistically significant.

## Results

All twelve subjects who entered the study (Figure 1) also completed all the four arms of the crossover study. There was no significant difference between treatment groups in baseline FEV_1 _prior to allergen; the values were 3.30 L after placebo, 3.40 L after Mometasone (400 μg), 3.38 L after Mometasone (800 μg) and 3.30 L after treatment with budesonide (800 μg).

### Early allergen response (EAR)

**Figure 2:**
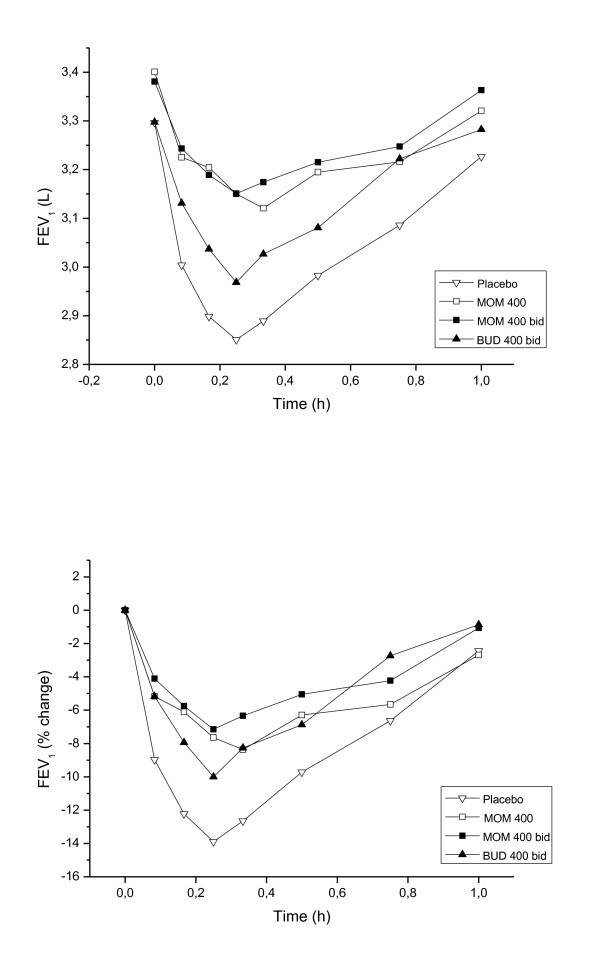
**Time course of FEV1 up to 1 h, % of baseline, and absolute FEV1, after inhaled allergen exposure during the different treatments arms (placebo, mometasone 400 μg, mometasone 800 μg and budesonide 800 μg daily)**.

Allergen challenge during the placebo treatment produced an immediate reduction in FEV1 reaching -13.9% at 15 min (Figure 2). The AUC for the EAR was 3.06 after placebo, 3.25 after the lower dose of mometasone, 3.26 after the higher dose and 3.15 after budesonide. Both concentrations of mometasone showed a significant difference in AUC EAR in comparison with placebo (P = 0.026, P = 0.017), but the effect of budesonide treatment was not statistically significant (P = 0.150). The maximum mean drop in FEV1 during the EAR was 8.37% after 20 min at the low dose and 7.15% after 15 min at the high dose of mometasone. The corresponding FEV1 value in the budesonide group was 10.0% after 15 min. The maximal drop in EAR was significantly less after the higher dose of mometasone vs budesonide (P = 0.02), but not significantly between the lower dose of mometasone vs budesonide (P = 0.06). There was no statistical difference between the effects of two different concentrations of mometasone on the EAR.

### Late asthmatic response (LAR)

**Figure 3:**
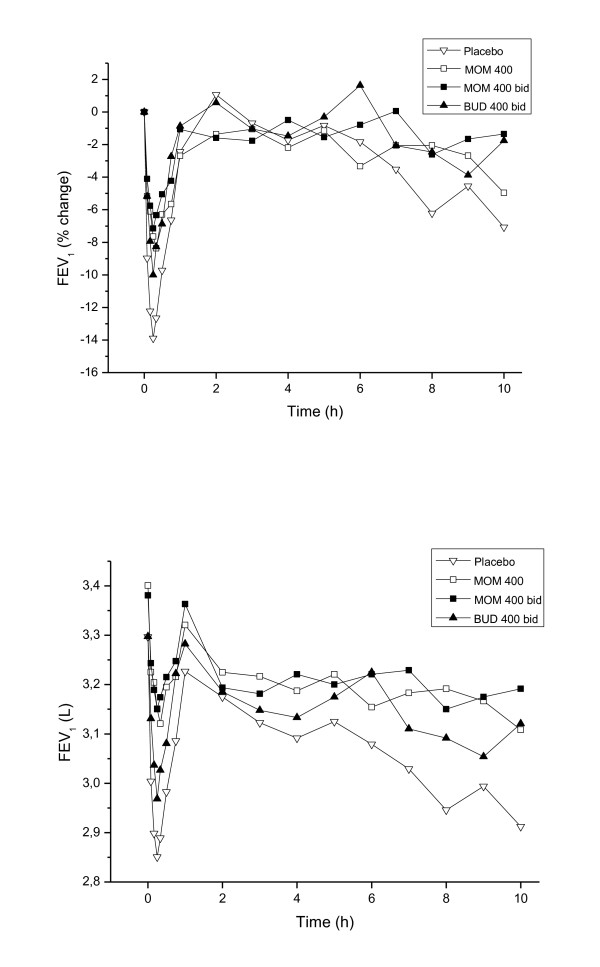
**Time course of FEV1 up to 10 h after inhaled allergen exposure during the different treatments arms (placebo, mometasone 400 μg, mometasone 800 μg and budesonide 800 μg daily)**.

Allergen challenge during the placebo treatment produced a late reduction in FEV_1 _reaching a maximum of 7.1% at 10 h. The AUC for the LAR between 1 to 10 h was 3.03 L after placebo, 3.18 L after the low dose of mometasone, 3.19 L after the high dose and 3.13 L after budesonide (Figure 3). The higher dose of mometasone showed a significant change in AUC of the LAR in comparison with placebo (P = 0.04), but the two other treatments did not significantly change the LAR, although tendencies were observed (mometasone 400 μg: P = 0.065, budesonide: P = 0.076). During mometasone treatment, the maximum late drop in FEV_1 _was 5% at 10 h during treatment with the low dose and 2.6% after 8 h during treatment with the higher dose. The corresponding FEV_1 _value in the budesonide group was 3.9% after 9 h. There were no significant differences among the different treatments for the LAR.

### Methacholine responsiveness

Twenty-four hours after allergen challenge the methacholine PC20 was 6.3, 11.1, 16.7 and 9.9 mg/ml during placebo, 400 μg mometasone, 800 μg mometasone and 800 μg budesonide treatment respectively. The result in all three treatment groups was statistically different from placebo. The low and high doses of mometasone reduced methacholine responsiveness with 2.6 and 2.8 doubling doses respectively in relation to placebo treatment (P = 0.004, P = 0.011), and the treatment with budesonide resulted in a doubling dose shift of of 2.4 vs placebo (P = 0.005). No significant differences were observed among treatments.

### Eosinophils

**Figure 4:**
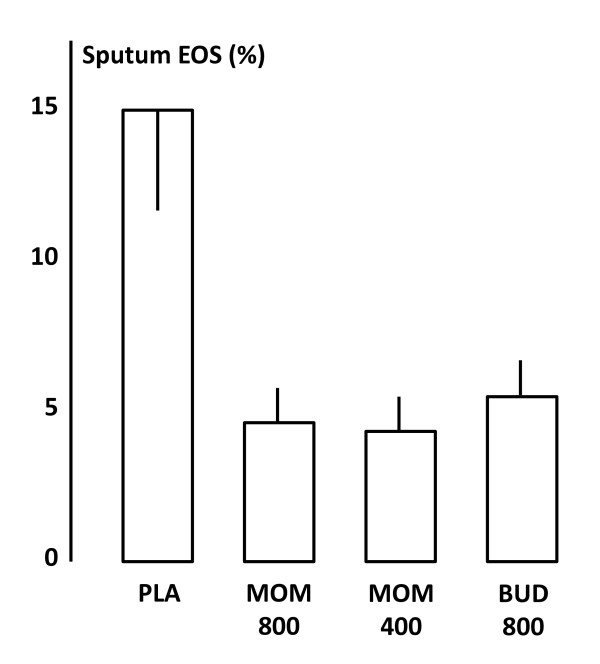
**Sputum eosinophils (%) 24 h after inhaled allergen exposure during the different treatments arms (placebo, mometasone 400 μg, mometasone 800 μg and budesonide 800 μg daily)**.

Sputum and blood were collected for the evaluations of eosinophils after allergen. There was a reduction of sputum eosinophils in all active treatment groups at 24 h after allergen provocation (Figure 4). The percentage of eosinophils in sputum was 14.6 ± 2.9 after placebo, 4.9 ± 1.6 after 400 μg mometasone, 4.8 ± 1.6 after 800 μg mometasone and 5.6 ± 1.7 after budesonide. Sputum-eosinophils were significantly decreased in all three treatment arms in comparison with placebo (p < 0.05), but no significant differences were observed among the active treatments. The number of blood eosinophils tended to be reduced in comparison with placebo after mometasone and budesonide both at 7 and 24 h (data not shown), but there were no differences observed among treatments.

### Urinary analysis

The detailed results of efficacy and urinary cortisol and other efficacy parameters are listed in Table 2. The 24 h urinary level of cortisol concentration was 215 ± 74, 179 ± 16, 135 ± 14, and 201 ± 38 nmol/L after treatment with placebo, 400 μg mometasone, 800 μg mometasone and 800 ug budesonide respectively. In the higher dose group of mometasone the cortisol level was significantly reduced (P < 0.001). A significant difference was also observed between the two dose levels of mometasone (P = 0.003), but not between the high dose of mometasone and budesonide (P = 0.07).

**Table 2:** Comparison between treatments

Analysis Means (SD)	Placebo	Mometasone 400 μg	Mometasone 2 × 400 μg	Budesonide 2 × 400 μg
Sputum-EOS (%)	14.60 (9.49)	4.94 (5.45)	4.83 (5.48)	5.66 (5.55)

B-EOS (10^9^/L)	0.32 (0.20)	0.22 (0.12)	0.18 (0.08)	0.20 (0.13)

S-ECP (μg/L)	13.24(13.56)	10.21 (8.80)	8.35 (7.97)	8.83 (8.39)

PC20 (mg/ml)	6.30 (10.77)	11.12(10.56)	16.72 (18.33)	9.88 (11.34)

U-Cortisol (nmol/L)	214.55 (74.19)	179.20 (55.80)	135.11 (49.39)	204.08 (130.45)

U-Creatinine (mmol/L)	12.91 (3.59)	13.86 (3.61)	12.90 (3.42)	12.92 (3.27)

## Discussion

This cross-over double blind placebo controlled study shows that one week treatment with both Mometasone 400 and 800 μg and Budesonide 800 μg affected physiological early and late asthmatic responses induced by inhaled allergen provocation, as well as inflammatory markers such as sputum eosinophils. The data suggests that Mometasone was more effective in attenuating the early asthmatic response than budesonide was at the same microgram daily doses. Furthermore, methacholine responsiveness 24 h after allergen provocation was significantly improved with all treatments. In parallel, effects of the inhaled glucocorticoids on serum cortisol was evaluated to determine airway effects vs systemic effects, showing only significant reduction of this parameter with the higher dose of mometasone.

This direct comparison between mometasone and budesonide suggests very similar inhibitory effects on allergen induced physiological responses in asthmatic patients with these two inhaled glucocorticoids, although mometasone at the higher dose cause a significantly greater inhibition of the early asthmatic response. The reduction of both the early and the late asthmatic response with the treatments was similar to what can be observed with other inhaled glucocorticoids [6,8,16]. It must be recognized that our main aim was to relate airway effects vs systemic effects, and therefore quite high doses of inhaled glucocorticoids were tested. Thus, it may be difficult to compare the exact potency of these drugs, as no full dose response could be performed with this complicated provocation protocol. In fact, such a full dose-response evaluation has not been reported with any inhaled glucocorticoid on allergen-induced asthma responses to date.

The three different active treatments all significantly and strongly reduced the sputum eosinophilia observed 24 h after airway allergen exposure, with no differences observed between them. This argues that both drugs and the doses tested are sufficient to efficiently attenuate the inflammatory response associated with the late asthmatic response, as has been previously documented [2,9,18]. The exact mechanism by which inhaled glucocorticoids have such pronounced effect on eosinophilic inflammation are not clear, although inhibitory effects on T-lymphocyte responses may be crucial [19].

Methacholine responsiveness, measured 24 h after the allergen provocation, was significantly attenuated by both doses of mometasone, as well as the single studied dose of budesonide. Importantly, this attenuation of bronchial hyperresponsiveness has two components to it. Firstly, the inhaled glucocorticoid significantly improves baseline bronchial hyperresponsivness [20,21], and secondly, it is also known that inhaled glucocorticoids can attenuate the increase in hyperresponsiveness induced by allergen exposure [15,16,22,23]. Thus, the attenuation of hyperresponsiveness observed in the current study is a composite effect of the inhaled glucocorticoid therapy on general bronchial hyperresponsiveness and the increased hyperresponsivness induced by the experimental allergen exposure. It is unlikely that the two studied inhaled glucocorticoids have fundamentally and mechanistically different effects on these separate explanations for the methacholine responsivenss value measured 24 h after allergen exposure.

With both doses of mometasone, and the single dose of budesonide studied, pronounced and highly significant inhibitory effects on allergen-induced sputum eosinophils were observed, and no dose-related effects of mometasone were obvious. This argues that the doses of inhaled glucocorticoids used in the current study was quite high for attenuating sputum eosinophils, and arguing that the anti-inflammatory efficacy of the doses studied were high.

Quite high doses of inhaled glucocorticoids were studied, to add the potential of studying also systemic effects of the two different inhaled glucocorticoids. Furthermore, we decided to study two doses of mometasone, to allow for documentation of some dose-related effects on either anti-asthma effects or systemic effects. Indeed, some differences in both airway and systemic effects were observed, with greater inhibition on the early asthmatic response with the higher dose of mometasone. However, in parallel a slightly increased effect on 24 h urinary cortisol was observed, which thus means that the higher dose of mometasone also expressed some systemic effects. Thus, the additive effect with the higher dose of mometasone was associated with greater systemic effect, arguing against any major difference in therapeutic ratio between the two different inhaled glucocorticoids.

In summary, both mometasone and budesonide at the doses studied (daily doses 400 and 800 μg for mometasone and 800 μg for budesonide) are highly effective in attenuating allergen-induced immediate airway responses, and strongly attenuate eosinophilic inflammation in the airways. Increased efficacy in the airways with the higher dose of mometasone was associated with a slightly higher degree of systemic effects, suggesting that increasing the dose is not necessarily associated with an improved therapeutic ratio. Inhaled glucocorticoids are efficient anti-inflammatory treatments in allergic asthma, and documenting any key differences in therapeutic effects and therapeutic ratio may deem difficult.

## Abbreviations

EAR: Early asthmatic responses; ECP: Eosinophil Cationic Protein; FEV1: Forced expiratory volume over 1 s; LAR: Late asthmatic responses; PC20: Provocative concentration changing FEV1 by 20%; AUC: Area under the plasma concentration - time curve

## Competing interests

JL has received honoraria for lectures and consultations from AstraZeneca, GlaxoSmithKline, MSD, Novartis, Oriel pharmaceuticals, Schering-Plough and UCB. PA is currently an employer of ALK Abelló, and has previously held an employment with GSK Sweden AB. MP has no reported conflict of interest.

## Authors' contributions

JL conceived and coordinated the study. MP and PA performed the study, and participated in the analysis and interpretation of the data together with JL. JL authored the bulk of the manuscript, with significant input from MP and PA. All authors have read and approved the final manuscript.
